# *ama1* Genes of Sympatric *Plasmodium vivax* and *P. falciparum* from Venezuela Differ Significantly in Genetic Diversity and Recombination Frequency

**DOI:** 10.1371/journal.pone.0003366

**Published:** 2008-10-10

**Authors:** Rosalynn L. Ord, Adriana Tami, Colin J. Sutherland

**Affiliations:** Department of Infectious & Tropical Diseases, London School of Hygiene & Tropical Medicine, London, United Kingdom; University of California Los Angeles, United States of America

## Abstract

**Background:**

We present the first population genetic analysis of homologous loci from two sympatric human malaria parasite populations sharing the same human hosts, using full-length sequences of *ama1* genes from *Plasmodium vivax* and *P. falciparum* collected in the Venezuelan Amazon.

**Methodology/Principal Findings:**

Significant differences between the two species were found in genetic diversity at the *ama1* locus, with 18 distinct haplotypes identified among the 73 *Pvama1* sequences obtained, compared to 6 unique haplotypes from 30 *Pfama1* sequences, giving overall diversity estimates of *h* = 0.9091, and *h* = 0.538 respectively. Levels of recombination were also found to differ between the species, with *P. falciparum* exhibiting very little recombination across the 1.77kb sequence. In contrast, analysis of patterns of nucleotide substitutions provided evidence that polymorphisms in the *ama1* gene of both species are maintained by balancing selection, particularly in domain I. The two distinct population structures observed are unlikely to result from different selective forces acting upon the two species, which share both human and mosquito hosts in this setting. Rather, the highly structured *P. falciparum* population appears to be the result of a population bottleneck, while the much less structured *P. vivax* population is likely to be derived from an ancient pool of diversity, as reflected in a larger estimate of effective population size for this species. Greatly reduced mosquito transmission in 1997, due to low rainfall prior to the second survey, was associated with far fewer *P. falciparum* infections, but an increase in *P. vivax* infections, probably due to hypnozoite activation.

**Conclusions/Significance:**

The relevance of these findings to putative competitive interactions between these two important human pathogen species is discussed. These results highlight the need for future control interventions to employ strategies targeting each of the parasite species present in endemic areas.

## Introduction

*Plasmodium vivax* infection causes 132–391 million cases each year worldwide, with a significant, but under-reported burden of severe malaria requiring hospitalisation [1]. Vivax malaria is endemic in both tropical and some temperate regions and it is estimated that 2.6 billion people are at risk from infection each year [2]. *P. vivax* is sympatric with *P. falciparum* in many regions often with *P. ovale* and/or *P. malariae* also [3]. The majority of laboratory and field studies of human malaria have been concerned with *P. falciparum*, due to the development of methods for continuous *in vitro* culture of this species, and the large number of genetic markers available [1], [4]. However the current elucidation of the *P. vivax* genome, and the recent development of improved *in vitro* drug sensitivity testing protocols [5] are cause for optimism regarding future research into this important human pathogen.

Control regimes targeting any particular malaria species must also consider knock-on effects this may have on other sympatric *Plasmodium* species. The effects a circulating patent species may have on liver-stage or sub-patent blood-stage infection by other species remains unclear. Working in Vanuatu, Maitland et al. [6] found that although the individual prevalence of *P. vivax* and *P. falciparum* fluctuated, the overall burden of malaria remained constant, suggesting that there was direct interaction between the two species. In regions where more than one species co-circulate, it has also been observed that fewer than expected mixed infections occur [6], [7], [8]. A suggested explanation for these observations is that an erythrocytic *P. falciparum* infection actively suppresses subsequent *P. vivax* infections that emerge from the liver [7] through non-specific effectors such as cytokines, and cross-species immune responses [7], [9]. When this suppression is lifted, either through drug treatment or host-mediated responses, there can be a swift expansion of *P. vivax* within the host [10]. Although a particular species, such as *P. falciparum*, may be reduced in prevalence or even eradicated in a particular setting, other malaria species, such as *P. vivax*, may survive the intervention through the persistence of dormant liver-stage hypnozoites, and subsequently proliferate through the human population to occupy the niche vacated by the targeted species. Thus although mortality due to *P. falciparum* may be reduced by such an intervention, malaria morbidity may continue; merely the species-specific dynamics within the region have been altered. In the light of drug resistance in *P. vivax*, [11], [12] and also possibly *P. malariae* [13], [14], control measures for the future that include novel drug and vaccine development should therefore consider all local sympatric *Plasmodium spp.* [3], [15].

To be effective in areas with multiple *Plasmodium* species, vaccine-based interventions would ideally generate protective responses against clinical episodes from each species present. Much vaccine development has focused on those antigens expressed during the merozoite stage, the form that invades circulating erythrocytes and reticulocytes during clinical stages of infection. Merozoite antigens are under considerable investigation as potential vaccine candidates despite extensive sequence variation within and between populations, as there is some evidence that antibodies raised to one variant are cross-reactive with other variants from the same species [16], [17]. Whether this cross-reactivity also indicates cross-isolate protection in subsequent infection with different variants is not known. A current vaccine candidate for both *P. falciparum* and *P. vivax* is the merozoite-expressed apical membrane antigen (AMA1). Widespread variation of the genes encoding AMA1 proteins from different populations and in more than one species have been reported, providing evidence that this gene, *ama1*, is under significant selection pressures in natural populations [15], [18], [19]. A number of studies have investigated polymorphism within *Pfama1* to assist development of anti-AMA1 vaccines able to elicit broad protective responses [18], [20], and this has lead to the development of multiple *Pfama1* antigens for simultaneous vaccination in order to overcome this diversity [17]. Analyses of diversity have also been carried out *Pvama1*, but these have used parasite isolates collected at hospital clinics, rather than cross-sectional *P. vivax* population samples [3], [15], [21]. There are no studies that examine *ama1* diversity simultaneously in both species in a setting where *P. vivax* and *P. falciparum* are sympatric.

We have been engaged in analysis of genetic diversity in malaria parasite populations circulating among forest-dwelling communities along the Padamo River basin in southern Venezuela. These studies have shown that the *P. falciparum* population in this area exhibits inter-genic linkage disequilibrium, and is dominated by a small number of multi-locus genotypes, whereas the *P. vivax* population appears more diverse [22], [23], [24]. Here we present full-length sequence analysis of *Pvama*1 and *Pfama*1 from cross-sectional surveys of malaria parasites co-circulating among the human population between 1995 and 1997. We use these homologous sequence datasets to directly compare genetic structure between the two parasite populations, to contrast selective forces acting upon the two genes, and to examine any evidence of direct interplay between the two species in this setting of mesoendemic, sympatric transmission among two geographically defined parasite populations sharing the same human hosts.

## Materials and Methods

Ethical approval for the original survey was obtained from the Ethics committee of the Venezuelan Ministry of Health, and the Ethics Committee of the London School of Hygiene & Tropical Medicine in 1995. The study protocol was also approved by CAICET (Centro Amazónico de Investigación y Control de Enfermedades Tropicales); the Dirección de Malariologa y Saneamiento Ambiental and the Regional Health Service, each institution providing a written statement of approval and support. Oral and written informed consent was obtained from the community leaders once the study was explained to the assembled community population, one community at a time.

### Sample collection

Between October 1995 and November 1997, blood samples and census data from all individuals in nine villages along the Padamo River, Amazon Basin, Venezuela, were collected in each of two cross-sectional surveys [23]. Due to the low *P. falciparum* prevalence found in 1997, the boundaries of the survey were extended in the second survey to include an additional large village. More than 90% of the surveyed population gave informed consent to participate: 708 individuals in 1996 and 945 in 1997. Blood samples from all participants were obtained for microscopy and collected onto filter paper for malarial genetic analyses. Individuals shown to have malaria parasites were treated accordingly, as described [23].

### DNA extraction

DNA was extracted from all available filter paper blood-spots corresponding to slide-positive samples as previously described [25]. Sufficient *P. falciparum* DNA samples for amplification and sequencing were not available from the 1997 survey. Samples that did not easily or consistently amplify were re-extracted using a commercially available kit (Qiagen) and this DNA source was used instead.

### *Pvama1* and *Pfama1* amplification and sequencing

*Pvama1*-specific primers were designed based on the published *P. vivax* sequence (GENBANK accession L27504) to amplify 1663 bp in a nested PCR approach. Previously published *Pfama1*-specific primers [19] were used in a nested PCR to amplify 1770 bp of the *Pfama1* gene. PCR positive samples were re-amplified in a 50 µl nest 2 reaction which was prepared for direct sequencing by ethanol precipitation into 20 µl. New sequencing primers for *Pvama1* were designed based on L27504. Previously published sequencing primers for *Pfama1* [19] were used in conjunction with amplification primers to determine full-length sequences (Perkin-Elmer BigDye 3.1). Sequencing reactions were cleaned by precipitation in 0.3 M NaAc, 125 mM EDTA and 2.2 volumes of ethanol, and analysed on an ABI prism 3730 automated capillary sequencer. Amplification and sequencing primers used in this study are presented in Table 1, with the PCR conditions used.

**Table 1:** PCR primers and conditions.

	Nest 1		Nest 2	
*Pvama1* 1663 bp	*Pvama*1_F16	5′ gcg gtt act tcc acc cc 3′	*Pvama*1_F29	5′ gca aac caa atc gct gcc 3′
	*Pvama*1_R613	5′ gcg tgg tgt ggg agg ccc 3′	*Pvama*1_R598	5′ gcc tcg ggg tcg agc atc tcg tc 3′
	Nest 1 PCR:	94°C^3 min^×1 cycle, (94°C^1 min^−46°C^1 min^−72°C^2.5 min^)×40 cycles, 72°C^10 min^×1	Nest 2 PCR:	94°C^3 min^×1 cycle, (94°C^1 min^−57°C^1 min^–72°C^2.5 min^)×40 cycles, 72°C^10 min^×1
	Fwd sequencing:		Rev sequencing	
	*Pvama*1_F142	aga att cca gct gga aga tg	*Pvama*1_R138	ttc cac ttc tgc atc ttc ccc
	*Pvama*1_F301	cgt aaa aat tta gga aac gcc	*Pvama*1_R293	aca ttt ttg ctc aaa tac acc
	*Pvama*1_F441	ccc ctg cag cat ata taa aga c	*Pvama*1_R442	ggg gaa atc ccg gtc tac ttc
*Pfama1* 1770 bp	*Pfama*1_F5*	5′ tgc gta tta tta ttg agc 3′	*Pfama*1_F10*	5′ gag cgc ctt tga gtt tac 3′
	*Pfama*1_R613*	5′ gtg ttg tat gtg atg ctc 3′	*Pfama*1_R598*	5′ gcc tca gga tct aac att tca tc 3′
	Nest 1 PCR:	94°C^3 min^×1 cycle, (94°C^1 min^−46°C^1 min^−72°C^2.5 min^)×40 cycles, 72°C^10 min^×1	Nest 2 PCR:	94°C^3 min^×1 cycle, (94°C^1 min^−60°C^1 min^−72°C^2.5 min^)×40 cycles, 72°C^10 min^×1
	Fwd sequencing:		Rev sequencing:	
	*Pfama1*_F143	gac ttc cat cag gga aat gtc c	*Pfama1*_R188	gag gtt ctg ttg gag gaa aag c
	*Pfama1*_F312	cgg att atg ggt cga tgg aaa ttg	*Pfama1*_R350	tta ggt tga tcc gaa gca ctc aa
	*Pfama1*_F485	gac agt tta aaa tgc cca tgt gc	*Pfama*1_R492	cac atg ggc att tta aac tgt c

* (Polley & Conway, 2001).

### Editing and assembling of sequence products

Each sequence from the same isolate was assembled and initially edited into a full-length contig with at least double coverage using SeqMan™II, and EditSeq™ (DNAStar Inc, Madison, WI). Secondary editing was done to confirm polymorphisms only occurring once (singletons) within the population using MegAlign™ (DNAStar Inc). Sequencing was repeated to confirm singletons and for isolates that did not have full-length double reads. A single *ama1* contig could not be determined for two isolates (one *P. falciparum* and one *P. vivax*). DNA was re-extracted and each was fully processed independently. In both cases, the two DNA extractions each yielded two different sequences that were already represented in the population, making them unlikely to be PCR artefacts, but rather indicating the infections were comprised of more than one genotype of that respective species. Confirmed single genotype data was exported as a PAUP alignment for statistical analyses, computed using DnaSP v.4.10 software.

### Within population analyses

In the 1997 survey, an extra village to the south of the main survey region, Koshirowetheri, was included for data collection and contributed a further 250 participants. To analyse data between the two years on a like-for-like basis, sequences from Koshirowetheri were at first excluded. Sequences of *Pvama1* from the original survey region from 1997 and those from Koshirowetheri in 1997 were subsequently compared by tests of departure from neutrality and analysis of within-population diversity to identify any significant differences between them that would preclude all *Pvama1* sequences obtained in the 1997 survey being treated as a single dataset.

The binomial probability was used to test whether the number of different haplotypes, and the relative frequency of the most common haplotype in each case, was significantly different between the *Pfama1* and *Pvama1* datasets. As 6 distinct *Pfama1* haplotypes (successes) were described in 30 sequences (trials) the probability of success in this dataset is 0.20. The probability that the observed number of successes in the *Pvama1* dataset was within a 2-sided 95% confidence interval of 0.20 was then calculated. Similarly, as 20 of 30 *Pfama1* sequences were identical, we tested whether the frequency of the most common *Pvama1* haplotype was significantly different to 0.667.

Tests to determine any significant departure of variation from neutrality using Tajima's D [26] and Fu and Li's D* and F* [27] indices were performed in DNAsp v. 4.10 [28] on each gene population as a whole and via a sliding window approach. Tajima's D tests the departure from neutral by comparing the estimations of nucleotide diversity (θ) derived from the average pairwise diversity (π) and the total number of polymorphic sites. The Fu and Li tests identify departures from neutral patterns of nucleotide substitutions as deviations between the estimates of θ derived from the number of phylogenies compared to either the total number of mutations (D*) or the average pairwise diversity (F*). Analysis of recombination and linkage disequilibrium was performed to calculate the minimum number of recombination events within each of the *Pvama1* and *Pfama1* sequences [29] and to give an estimation of the recombination parameter, *C* [30]. The indices of linkage disequilibrium, D' [31] and R^2^ [32] were also determined and their relationship with distance between sites was plotted.

### Between population analyses

The Mc Donald-Kreitman test, using either *P. cynomolgi* (accession number X86099) or *P. knowlesi* (accession numbers AF298218, M58317, M61097) for outgroup comparisons with the *P. vivax* populations, and *P. reickenowi* (accession number AJ252087) for comparisons with *P. falciparum*, allows for determination of the ratios of synonymous and nonsynonymous changes between and within species. Between-population divergence of the *Pvama1* data presented here and previously published *Pvama1* data for Domain I (DI) from global sites [15], [33], [34], [35] was determined using the θ-estimator of Wright's fixation index (F_ST_) to determine the relative contribution of the differences observed between each population to the overall diversity seen using Arlequin v.3.11 software [36]. As only two previously published studies [15], [35] allow comparisons across the whole *Pvama1* ectodomain, F_ST_ values in our dataset were determined across the whole gene and for each of the domains separately. Mean F_ST_ values for the Venezuelan *Pfama1* sequences were compared with two published *Pfama1* data sets [18], [19] that each includes all three domains, allowing determination of mean F_ST_ values for the full gene and each of the three domains separately.

## Results

### Parasite samples

In the first survey, light microscopy determined 40 *P. vivax*, 32 *P. falciparum*, 16 *P. malariae* and 2 mixed infections (one *P. vivax* with *P. malariae* and one *P. vivax* with *P. falciparum*), out of 708 individuals. In the second survey of 945 individuals, microscopy determined 92 *P. vivax*, 12 *P. falciparum*, 20 *P. malariae* and 3 mixed infections of *P. vivax* with *P. malariae*. The overall malaria prevalence did not vary between surveys being 12.7% in 1995/6 and 13.4% in 1997. However, vivax malaria comprised a significantly greater proportion of malaria infections in the second survey (O.R. 3.39, 95% C.I. 1.83–6.29; P<0.0001), whereas falciparum was significantly less common (O.R. 0.180, 95% C.I. 0.079–0.394; P<0.0001). Full-length *ama1* contigs with double sequence reads were obtained for 73 *P. vivax* and 30 *P. falciparum* isolates (GenBank accession nos. EU346015-EU346087 for *Pvama1*, and EU332414-EU332443 for *Pfama1*). All sequences encoded cysteine residues in previously described positions. Neither *Pvama1* nor *Pfama1* populations contained sequences identical to their respective reference strains; Sal-1 (gene ID Pv092275 from http://www.PlasmoDB.org) and 3D7 (accession number XM_001347979).

### Within population analyses

Diversity among *Pvama1* sequences was first analysed within comparable groups: those from the 9 villages surveyed in 1995/6 (28 sequences), those collected from these 9 villages in the second survey (12 sequences), and sequences collected from Koshirowetheri village in the second survey (33 sequences). Diversity of the *Pfama1* gene was analysed in sequences from the 9 villages in the first survey only (30 sequences).

**Figure 1:**
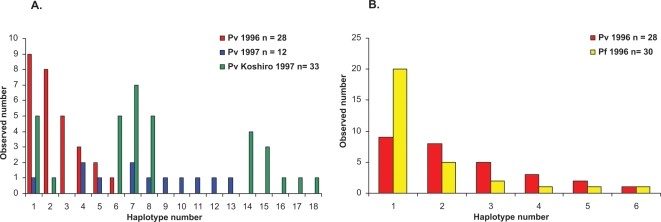
Graphical representation of the distributions of the *Pvama1* and *Pfama1* allele frequencies from the Venezuelan Amazon. A. Distribution of 18 *Pvama1* haplotypes across surveys in 1996 and 1997. Sequences from the additional village sampled in 1997 (Koshiro) are presented separately from those of the villages sampled in both surveys. B. Haplotype frequency distribution for *Pfama1* and *Pvama1* from the 1996 survey only.

Genetic diversity, as measured by *h*, is greater among the *Pvama1* sequences than among the *Pfama1* sequences (Table 2). This remains true when isolates from Koshirowetheri are excluded from the analysis, supporting the conclusion that there is a significant increase in the circulating diversity of *P. vivax* at the *Pvama1* locus between the two years (Figure 1). Of the 73 *Pvama1* sequences obtained, there were 18 unique haplotypes, compared to 6 unique haplotypes from 30 *Pfama1* sequences (2-sided binomial probability, P = 0.308). However, among the 30 *Pfama1* sequences 20 were of the most common haplotype, whereas among the 73 *Pvama1* sequences 15 were of the most common haplotype (P<0.001). The overall diversity estimates for *Pvama1* and *Pfama1* (*h* = 0.9091, *h* = 0.538 respectively) are consistent with our previous studies from this region using *rif*, *Pfglurp*, *Pfmsp1* and *Pfmsp2* genes for *P. falciparum*, and using *Pvmsp3α* for *P. vivax* [22], [23], and demonstrate greater diversity in the *P. vivax* population.

**Table 2:** Within-population analyses of *Pvama*1 and *Pfama*1 from the Venezuelan Amazon.

	*P. falciparum ama1*	*P. vivax ama1*		
	1996	Villages 1–9 in 1996	Villages 1–9 in 1997	1997 Koshirowetheri
Number of sequences	30	28	12	33
Number of haplotypes				
Whole gene	6	6	10	10
Domain I	5	5	6	9
Domain II	2	3	4	3
Domain III	3	2	3	2
Gene diversity, *h*				
Whole gene	0.54	0.80	0.97	0.89
Domain I	0.45	0.78	0.68	0.86
Domain II	0.37	0.58	0.74	0.48
Domain III	0.42	0.50	0.59	0.41
Tajima's D	1.73927*	1.00507**	−0.48752	1.82661
Fu and Li's D*	1.72279***	0.71243**	−0.53615	1.72312***
Fu and Li's F*	2.03574***	0.94666**	−0.59625	2.07362***
Recombination parameter, *C* (Between adjacent sites)	0.001 (0.000)	10.0 (0.0061)	14.7 (0.0090)	16.9 (0.0103)
Effective population size, *Ne* †	4.2×10^2^	4.2×10^6^	6.1×10^6^	7.0×10^6^
Minimum Recombination events, Rm	3	1	4	8

^*^ = 0.10>P>0.05; ^**^ = P>0.10; ^***^ = P<0.02.

† = Effective population size, *Ne*, = C/4*r*, where *r* is the recombination rate and *r* of *P. falciparum* is used for all, approximately 6×10^−7^, as the recombination rate for *P. vivax* has not yet been determined (Joy *et al.*, 2006).

**Figure 2:**
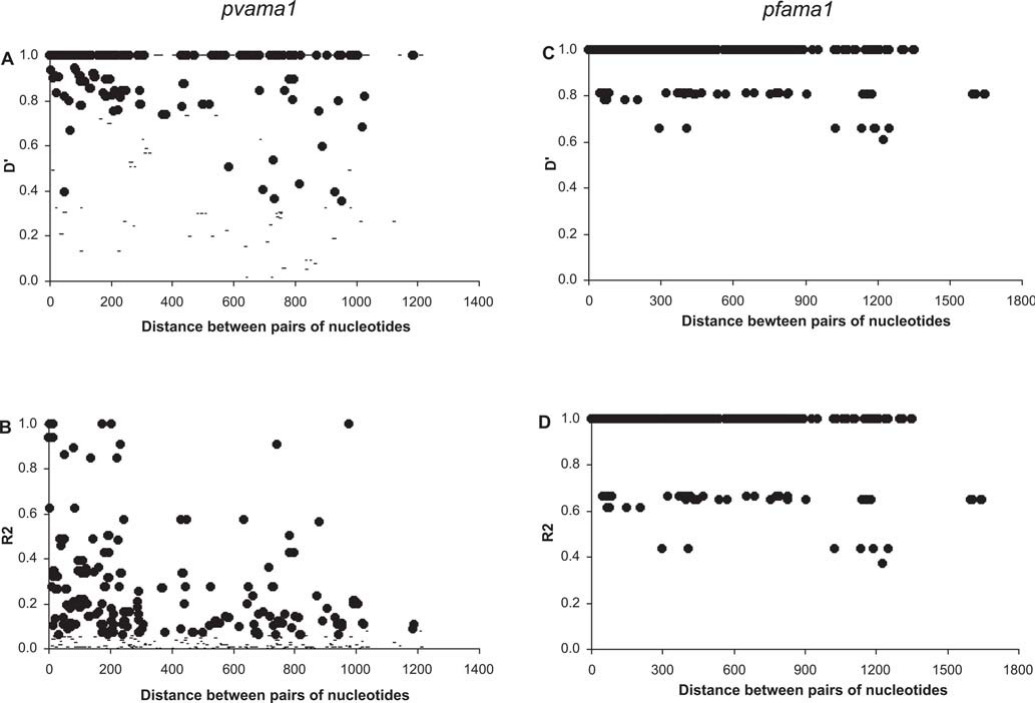
Linkage disequilibrium indices of *Pvama*1 and *Pfama*1. Linkage disequilibrium plots of D' and R^2^ for *Pvama1* (Graphs A and B) and *Pfama1* (Graphs C and D). Sites with significant linkage (P<0.05) are shown as solid circles; non-significant sites are shown as for *Pvama1* only. No non-significant sites for *Pfama1* were found.

The frequency of recombination within the *Pvama1* population is relatively high compared to the predicted frequency within the *Pfama1* population (10 minimum recombination events within 73 *Pvama1* sequences vs. 3 in 30 *Pfama1* sequences; Table 3), yet both of these are low compared to estimates from other regions. Among 23 *Pvama1* sequences from Sri Lanka, the estimated minimum number of recombination events was 9 [15]. For *Pfama1*, analysis of 50 isolates from Nigeria and 51 from Thailand provided estimates of the minimum number of recombination events of 25 and 16 respectively [18], [19]. Our linkage disequilibrium analysis indicates declining inter-site linkage with increasing nucleotide distance within the *Pvama1* sequences, in contrast to the significant linkage across the whole of the *Pfama1* sequences (Figure 2.). The very low value of the *Pfama1* recombination parameter, *C* = 0.001, and the observed maintenance of significant linkage suggests very little meiotic recombination occurs in this gene in our population, supporting the view that clonal propagation of *P. falciparum* has occurred over a significant period of time [23]. This is supported by our estimation of en effective population size for *P. vivax* that is 4 orders of magnitude higher than that for *P. falciparum* (Table 2).

**Table 3:** McDonald-Kreitman analyses of *Pvama*1 and *Pfama*1.

	Region	Polymorphic changes within species	Polymorphic changes within species	Fixed differences between species	Fixed differences between species	Neutrality index^1^	Fisher's exact test^2^
		Synonymous	Nonsynonymous	Synonymous	Nonsynonymous		
*P. vivax* vs	Whole	6	21	119	69	6.04	0.000095**
*P. cynomolgi*	DI	3	11	35	19	6.75	0.0057*
	DII	2	2	21	11	1.91	0.61
	DIII	0	2	12	10	-	0.48
*P. vivax* vs	Whole	9	22	130	82	3.88	0.00086**
*P. knowlesi*	DI	4	12	37	21	5.29	0.0095*
	DII	2	2	20	21	1.67	1.00
	DIII	1	2	15	3	10.00	0.13
*P. falciparum* vs	Whole	0	29	19	53	-	0.0013*
*P. reickenowi*	DI	0	14	4	10	-	0.098
	DII	0	5	3	4	-	0.20
	DIII	0	3	1	5	-	1.00

1 = Neutrality index indicates the extent to which the levels of amino acid polymorphism depart from the expected in the neutral model.

2 = Fisher's exact test.

*0.001<P<0.01; ** P<0.001.

**Figure 3:**
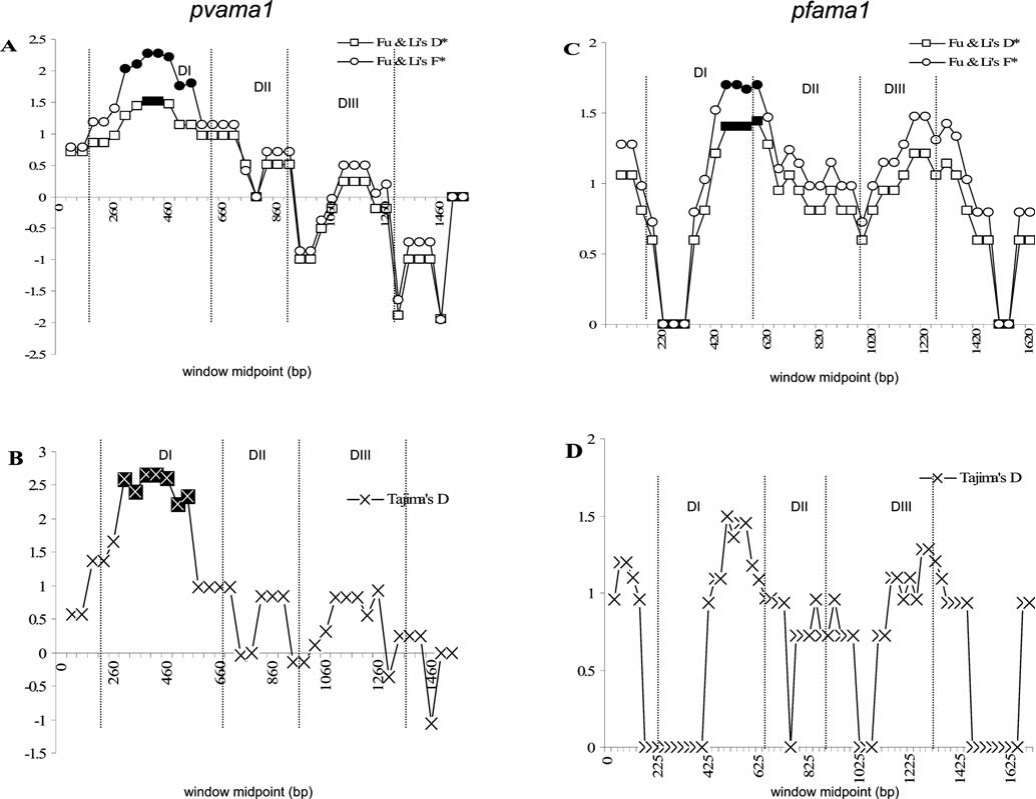
Sliding window plots of Tajima's D and Fu and Li's F* for *Pvama*1 and *Pfama*1. A and B – *Pvama1*; C and D – *Pfama1*. Midpoints where the sequence significantly departs from zero (P<0.05) are shown as solid points. Domains, DI, DII, DIII, are marked.

Evidence of selective signals in the two sequence sets was provided by tests for neutrality of polymorphisms. Across the two full-length genes, significant departures from neutrality were only found for *Pfama1*, with Fu and Li's D* and F* analyses providing parameter values of 1.72279 and 2.03574 (P<0.02 in each case) respectively. For *pvama1*, sequences from the 1997 survey gave significant values for Tajima's D and Fu and Li's F* across domain 1 only (data not shown). Sliding window analyses were also performed to identify any departures from neutral patterns of nucleotide substitution across smaller regions within both genes. Plots of each of these analyses, with the individual domains highlighted, are shown in Figure 3. As with other studies [15], [33], [34], a region of DI of *Pvama1* within this Venezuelan population shows a significantly positive departure from neutral substitution patterns, indicating that balancing selection may be maintaining alleles of this domain. However, unlike previous studies [15], no evidence was found of balancing or directional selection upon DII or DIII in *Pvama1* sequences from this Venezuelan population, and the values for these two domains are effectively zero (minimally positive and minimally negative in many cases). Like the *Pvama1* data, *Pfama1* showed a significant positive departure from neutrality in DI. The trend across the other two domains matches published Nigerian and Thai data of Polley et al. [18], [19], in that evidence of allele maintenance by balancing selection was stronger in DIII than within DII, although this was not significant in our study population.

### Between population analyses

We also examined the two sequence sets for evidence of selection using the McDonald-Kreitman test, which compares the relative frequency of non-synonymous nucleotide substitutions within the species, to the relative frequency of fixed differences between this and a related species. Data are summarised in Table 3, and support results from the tests of neutrality, as alleles within these Venezuelan *Pvama1* and *Pfama1* populations, particularly within DI, have significantly more non-synonymous substitutions than expected from comparison with related species. Thus diversifying selection is maintaining *ama1* allelic diversity in both *P. falciparum* and *P. vivax* in our population.

There are 12, 4 and 3 *Pvama1* haplotypes within this studied Venezuelan population across DI, DII and DIII, respectively. When this Venezuelan data is added to previously published data [15], [33], [34], [35], there are 90 described haplotypes across DI, 11 of which are unique to Venezuela; 8 haplotypes across DII, 1 unique to Venezuela; 4 haplotypes across DIII, none unique to Venezuela. This observed greater overlap of haplotypes across DII and DIII between samples from geographically diverse regions may be due simply to the scarcity of polymorphic sites in these smaller domains, exacerbated by the fact that only two other studies are available for comparison across these domains [15], [35]. Inter-population comparison of *Pvama1* sequences from all available studies shows that the majority of diversity is within each population and not among populations, as indicated by low F_ST_ values (Tables 4 and 5).

**Table 4:** Geographical differences in *Pvama1* sequences across DI only.

	Venezuela^a^														
Venezuela^b^	**0.140**	Venezuela^b^													
Venezuela^c^	**0.059**	0.824	Venezuela^1^												
		Sri Lanka^2^	**0.125**	Sri Lanka	(23)										
		Thailand^3^	**0.135**	**0.122**	Thailand	(8)									
		ADS^4^	**0.170**	**0.152**	**0.139**	ADS	(21)								
		Africa^4^	**0.134**	0.003	0.104	**0.156**	Africa	(5)							
		China^4^	**0.092**	**0.075**	0.020	**0.096**	0.047	China	(7)						
		India^4^	**0.085**	0.034	**0.081**	**0.126**	0.044	0.032	India	(18)					
		Indonesia^4^	0.072	0.043	0.059	**0.116**	0.050	0.000	*−0.011*	Indonesia	(5)				
		Morong^4^	**0.146**	**0.128**	**0.107**	**0.155**	**0.115**	**0.084**	**0.099**	0.078	Morong	(110)			
		Palawan^4^	**0.163**	**0.143**	**0.139**	*−0.012*	**0.146**	**0.091**	**0.117**	0.094	**0.155**	Palawan	(17)		
		PNG^4^	**0.107**	**0.097**	0.045	**0.120**	0.029	0.016	**0.050**	0.022	**0.102**	**0.112**	PNG	(23)	
		Solomons^4^	**0.131**	**0.123**	*−0.007*	**0.151**	0.097	0.048	**0.075**	0.051	**0.125**	**0.142**	0.009	Solomons	(7)
		Thailand^4^	0.074	*−0.008*	*−0.007*	**0.112**	*−0.020*	0.000	*−0.014*	*−0.034*	**0.078**	**0.102**	0.007	*−0.024*	Thailand (6)
		India (33)^5^	**0.109**	**0.066**	**0.089**	**0.137**	0.050	0.039	0.011	0.023	**0.109**	**0.128**	**0.054**	**0.083**	0.009

Pairwise F_ST_ values between Venezuelan sequences presented here and other sites only are highlighted. ^a^ = 1996 villages, N = 28; ^b^ = 1997 villages, N = 12; ^c^ = Koshirowetheri 1997 only, N = 33; ^1^ = all Venezuela data irrespective of village, N = 73; ^2^ = [15]; ^3^ = [33]; ^4^ = [34]; ^5^ = [35]; Sample sizes available are shown in parenthesis after each location; Significant difference (P<0.05) indicated by bold underline. Slightly negative numbers, indicated by italics, are biologically meaningless and are equivalent to F_ST_ = 0.000.

**Table 5:** Geographical differences in *Pvama1* and *Pfama1* sequences across all domains.

*P. vivax ama1* pairwise F_ST_ estimates	**Venezuela** **(73)** 1		*P. falciparum ama1* pairwise F_ST_ estimates	**Venezuela (30)** 2	
**Sri Lanka (23)** 2			**Nigeria (50)** 4		
Whole	**0.074**		Whole	**0.258**	
DI	**0.120**		DI	**0.265**	
DII	**0.185**		DII	**0.192**	
DIII	**0.322**	**Sri Lanka**	DIII	**0.240**	**Nigeria**
**India ** 3			**Thailand (51)** 5		
Whole (33)	**0.091**	**0.048**	Whole	**0.273**	**0.019**
DI (33)	**0.109**	**0.058**	DI	**0.286**	**0.039**
DII (34)	0.034	**0.223**	DII	**0.344**	**0.086**
DIII (34)	**0.349**	*−0.035*	DIII	**0.276**	**0.056**

Pairwise F_ST_ values between Venezuelan sequences and other sites are highlighted. Sample sizes are in parenthesis. ^1^ = all Venezuela *Pvama1*data from both 1996 and 1997; ^2^ = all Venezuela *Pfama1* data from 1996 only; ^2^ = [15]; ^3^ = [35] one sequence was ignored in the whole gene and DI analysis as it was not full length; ^4^ = [19]; ^5^ = [18]. Significant difference (P<0.05) indicated by bold text. Slightly negative numbers, indicated by italics, are biologically meaningless and are equivalent to F_ST_ = 0.000.

The number of *Pfama1* haplotypes observed in the Venezuelan dataset is 6, 2 and 3 across DI, DII and DIII, respectively. When added to previously published data from Nigeria and Thailand [18], [19], there are 58 haplotypes across DI, 5 of which are unique to Venezuela; 26 haplotypes across DII, none unique to Venezuela; 17 haplotypes across DIII, none of them unique to Venezuela. Pairwise inter-population comparisons show there is a significant diversity between population pairs, particularly across DI, with generally higher F_ST_ values than for *Pvama1* (Table 5).

## Discussion

We have analysed diversity in full length gene sequences encoding the apical membrane antigen (AMA1), from 73 *P. vivax* and 30 *P. falciparum* isolates collected in the same cross-sectional surveys of an isolated human population. To our knowledge, this is the first such detailed analysis of two homologous genes in sympatric *Plasmodium* parasite populations. The majority of people present at the time of the surveys agreed to provide blood samples for analysis, irrespective of malaria status, and so these surveys represent a good sample of both co-circulating *P. vivax* and *P. falciparum* [22], [23]. This is an important strength of our study, compared to analyses of symptomatic cases that have presented passively at health facilities with undefined catchment areas.

Previous studies had suggested that *P. vivax* and *P. falciparum* circulating in the Padamo exhibited very different population structures, but a direct test of this hypothesis required detailed analysis of homologous genes in the two species populations, so that the magnitude and effect of selection would be as similar as possible in the two datasets. AMA1 is a well-characterised parasite protein in both species, with homologues in all *Plasmodium spp*. examined, and is currently being tested as a vaccine candidate for *P. falciparum*. AMA1 contributes to the reorientation of merozoites, and is located at the tight junction during ingress of the erythrocyte. AMA1 also has a role in sporozoite invasion of hepatocytes [37], [38], [39], [40].

Genetic diversity was found to be significantly higher in the *P. vivax* population, and indices of intra-population recombination were significantly lower in the *P. falciparum* population. In contrast, indices of selective diversification on the two gene populations were similar, suggesting that differences in functional and immunological constraints upon them could not explain differences in gene diversity, and the frequency of recombination. We therefore conclude that whereas the *P. vivax* population is comprised of a diverse gene pool that frequently undergoes recombination, the *P. falciparum* population exhibits restricted diversity and appears to propagate most commonly by clonal expansion, with minimal opportunities for recombination. These findings are wholly consistent with the observation of stable multi-locus genotypes supported by high levels of inter-genic linkage disequilibrium among the *P. falciparum* population in the Padamo. As argued previously, this strongly suggests that this parasite population has passed through an evolutionary bottleneck which has both restricted genetic diversity and reduced opportunities for recombination within the population [23]. In contrast, we found no evidence that the *P. vivax* population has also passed through such a bottleneck. Evidence of a similar difference in structure between these two species has been observed in a study of 11 isolates of each from Papua New Guinea [41]. Interestingly, our earlier analysis of *var* gene sequences in the Padamo *P. falciparum* population indicated that these genes partially transcended the clonal structure observed at other loci, supporting the view that this gene family recombines, and evolves, at a higher frequency than do other loci in *P. falciparum* [42].

The majority of diversity seen among the Venezuelan *ama1* sequences is in DI of both *P. vivax* and *P. falciparum* and, in accordance with other global studies, this domain accounts for the majority of distinct haplotypes within a population. However, the polymorphisms generating this diversity do not demonstrate neutral patterns of substitution. The pattern of polymorphisms seen in DI show a significant positive departure from neutral, suggesting they are under biological functional constraints as well as being targets of host immune mechanisms. Evidence of such ‘balancing selection’ in DI of *ama1* across global isolates suggests this region is a target of natural host immunity, and supports the development of this domain as a potential vaccine target in both *P. vivax* and *P. falciparum*.

The importance of DII is less clear. In this Venezuelan population, there is no evidence to suggest balancing selection has contributed to the pattern of polymorphic substitution neither in DII nor in DIII of either species. Previous data confirms this finding for *Pfama1* [18], [19], but this is contradictory to available *Pvama1* data [15]. However, the *Pvama1* sequences from this smaller Sri Lanka dataset (N = 23) were derived from clinical malaria presentations, and it is unknown how those alleles seen compare to the full repertoire of alleles present within the circulating *P. vivax* population from that region. Such differences in sampling procedure may also explain the neutral pattern of substitution across DIII observed among the Venezuelan *Pvama1* sequences, in contrast to DIII from other regions.

Low sample numbers may have inhibited our analyses of the Venezuelan *Pfama1* sequences by reducing the power needed to identify significant changes or signals of selection beyond those identified in DI. Only 30 *Pfama1* sequences were obtained, all from the first survey in 1996. Yet, as only 33 individuals were slide positive for *P. falciparum* in this year (one of which was a mixed infection with *P. malariae*), under-representation is unlikely. The potential lack of power within *Pfama1* is shown in DIII. Although the trend follows that previously described, there is no significant evidence to conclude this domain is also under balancing selection.

There is a marked difference in the prevalence of *P. vivax* infections between the two surveys. The second survey in 1997 followed a period of low rainfall which effectively interrupted mosquito-borne transmission for that season [23]. *P. falciparum* prevalence (slide positive) dropped from 4.7% to 1.3% but *P. vivax* increased from 5.5% in 1996 to 9.8% in 1997. *P. malariae* prevalence remained similar (2.3% and 2.1% respectively) as did the overall burden of malaria −12.7% in 1996 to 13.44% in 1997. In the Venezuelan Amazon, both *P. vivax* and *P. falciparum* are transmitted by the predominant vector species, *Anopheles darlingi*, and an interruption of transmission would affect both parasite species equally.

We suggest the increase of prevalence of *P. vivax* regardless of reduced transmission is a result of new merozoites being released from dormant hypnozoites (relapse). In the presence of active *P. falciparum* infection, such emergent *P. vivax* blood-stages may be prevented from establishing an infection by cross-species regulation and suppression [7], [9]. The presence of circulating *P. falciparum* may have effectively limited *P. vivax* infection in the previous (wet) year, but during a season with greatly reduced transmission, these new merozoites are able to expand significantly within their individual hosts. It has usually been assumed that genotypes seen as a result of relapses will always be representative of those genotypes previously seen, i.e. those of a primary infection [43], [44]. If true, then even though the actual numbers of *P. vivax* increased from one survey to the next in this Venezuelan study, the genetic profiles between the two samples would be expected to be similar. We found a greater apparent diversity in *Pvama*1 sequences from the 1997 survey, with whole gene diversity *h* = 0.913 compared to *h* = 0.796 in 1996. This may be partially due to the lower number of isolates obtained in the first survey, and given the large effective population size we have estimated for *P. vivax*, we cannot be certain our two cross-sectional samples were sufficiently representative to permit comparison of *P. vivax* diversity between surveys. Pairwise F_ST_ values show small but significant differences between *Pvama1* sequence sets between these two years across the whole gene (9.1%) and across the polymorphic region of DI only (6.3%). We cannot determine whether any apparent increase in diversity in the second survey is due to relapse from heterologous hypnozoites, as we did not successfully amplify *Pvama1* from any sample pairs of individuals that were *P. vivax* positive in both 1996 and 1997 surveys (there were 3 such people). Further work on *pvama1* diversity in larger samples from this population would be required to compare with recent findings from Thailand, India and Myanmar which suggest that relapse of *P. vivax* in these regions is usually from activation of heterologous hypnozoites [45].

Of the four *Plasmodium* species that infect humans, *P. vivax* and *P. falciparum* are the two that are most often sympatric. Despite this, control regimes have usually targeted only *P. falciparum* in isolation, and in many cases the contribution of *P. vivax* to the local and global malarial burden has been ignored [1]. Substantial differences in the level of genetic diversity between these two species in the Padamo are consistent with one of them, *P. falciparum*, having passed through an evolutionary bottleneck either at the time of the introduction of this species to Venezuela from Europe and Africa or due to more recent constraints on recombination, whereas the *P. vivax* population displays evidence of a distinct recent evolutionary history in which diversity is maintained, and recombination is common. Whereas the former species displays characteristics of an epidemic population structure, *P. vivax* exhibits a structure consistent with that of an established endemic pathogen. Our data thus demonstrate that these two human pathogens adopt different strategies to survive in this setting of mesoendemic transmission in the Venezuelan Amazon, and as a result differ in their susceptibility to changing environmental conditions, and seasonal fluctuations in vector abundance. Future control regimes and interventions therefore need to include strategies that will target each of the parasite populations present, and so aim to reduce the overall malarial burden.
